# Transition From Nasogastric Tube to Oral Feeding: The Role of Parental Guided Responsive Feeding

**DOI:** 10.3389/fped.2019.00190

**Published:** 2019-05-09

**Authors:** Iris Morag, Yedidya Hendel, Dalia Karol, Ronny Geva, Strauss Tzipi

**Affiliations:** ^1^Sackler School of Medicine, Tel Aviv University, Tel Aviv, Israel; ^2^Chaim Sheba Medical Center, The Edmond and Lily Safra Children Hospital, Ramat Gan, Israel; ^3^Department of Psychology, Bar Ilan University, Ramat Gan, Israel; ^4^The Gonda Multidisciplinary Brain Research Center, Bar Ilan University, Ramat Gan, Israel; ^5^Faculty of Medicine, University of Ottawa, Ottawa, ON, Canada

**Keywords:** preterm infant, oral feeding, responsive feeding, weight gain, nasogastric tube

## Abstract

**Background and Objective:** Strategies to transition preterm infants from tube to oral feeding vary greatly and the transition may take days to weeks. The study objective was to evaluate the effect of parental guided responsive feeding (PGRF) on this transition.

**Methods:** We conducted a randomized controlled trial on infants born at <32 weeks gestation. The PGRF intervention was performed by parents, and included feeding intervals and volumes which were guided by the infants' behavioral cues of hunger and satiety. If a minimum volume was not taken orally, an intermediate volume was supplemented via nasogastric tube. The control group was traditionally fed (TF), with pre-planned volumes of intake and at given scheduled intervals.

**Results:** The study comprised 67 infants (PGRF 32, TF 35). PGRF infants reached full oral feeding within less days (median 2 vs. 8 days, *p* = 0.001), at an earlier age (median 34.28 vs. 35.14 weeks, *p* < 0.001), returned to baseline weight gain at 35 weeks (1.77 ± 0.70 vs. 1.25 ± 0.63 g/kg/day, *p* = 0.002), were discharged earlier (36.34 ± 0.6 vs. 36.86 ± 0.9 weeks, *p* = 0.001), were more likely to be fed by their parents (*p* < 0.001), and experienced less apnea/bradycardia events at 34 weeks (median 3.5 vs. 9 per week *p* = 0.047) compared to the TF infants. The regression model demonstrated that independent variables predicted 43.7% of the variance of time to full oral feeding [*F*_(9, 65)_ = 4.84 *p* < 0.001]. The only significant variable was feeding group (*B* = −6.43 *p* < 0.001); The PGRF infants were more likely to reach full oral feeding earlier.

**Conclusion:** PGRF is safe, and associated with short-term advantages, higher parental engagement, and earlier discharge.

**Clinical Trial Registration:** Identifier: SHEBA-12-9574-IM-CTIL; “Adjusted Individual Oral Feeding for Improving Short and Long Term Outcomes of Preterm Infants.”

## Introduction

Physiologically stable preterm infants, are generally transitioned from tube feeding to oral feeding at 32–34 weeks gestational age. This transition may take days to weeks (1). Success in this transition, defined as adequate intake for growth and maintenance of physiologic stability, depends on several factors: (a) the infant's neurological and physiological maturity, namely, the infant's ability to remain engaged in feeding, organize oral-motor functioning, co-ordinate swallowing with breathing, and maintain physiologic stability (2, 3); (b) the caregiver's ability to co-regulate the infant during feeding, namely, to timely recognize and respond to the infant's behavioral and physiological cues, aiming to prevent physiological de-compensation and repeated stress (1); and (c) the NICU's approach toward feeding. The NICU's approach toward feeding can be varied among hospitals. In many NICUs, the transition to oral feeding is regarded as a simple technical task, and is often delegated to the least experienced professional, such as a new nurse or even a volunteer (1). Moreover, traditional feeding (TF), which is also known as volume-driven feeding, is still commonly practiced in many NICUs. TF involves administering a pre-planned volume of food at given scheduled intervals, regardless of infant cues. In order to successfully administer a pre-planned volume, various maneuvers are used, such as twisting the bottle or moving the nipple in and out (4). Such strategies often disregard the infant's coordinated feeding behaviors (5, 6), and overlook behavioral cues, since the primary goal of TF is to finish the pre-planned volume of food. As a result of this feeding method, repeated desaturations and aspirations may occur, resulting in physiologic decompensation and repeated stress (7, 8). These repeated stressful experiences during the time a preterm infant learns to feed have been associated with a long-lasting aversion toward feeding. This aversion to feeding has been demonstrated to last until those preterm infants reach 6 years of age (4, 9–14).

Recently, a new approach to feeding preterm infants, known as a “responsive,” “sensitive,” “infant-driven,” or “cue-based” feeding, has been studied (15). According to this approach, the caregiver's ability to understand and respond to the infant's behavioral communication, especially during feeding, plays a key role in the infant's ability to feed effectively. When the infant's behavior is perceived as meaningful (i.e., having communicative intent), the focus changes from a volume-driven approach to a co-regulated approach, in which the infant guides the caregiver (10). Theoretically, feeding skills that develop at an infant's own pace may be associated with better self-regulation during feeding and this will facilitate oral feeding progression (7). This responsive method of feeding also enhances parents' experiences of nurturing their child and can create a more enjoyable, pleasurable, and satisfying experience for both infant and parent (7, 10). A recent Cochrane meta-analysis, which enrolled nine randomized controlled trials (*n* = 593), reported on early neonatal outcomes among preterm infants subjected to responsive feeding vs. TF protocols (16). Infants subjected to the responsive feeding protocol achieved full oral feeding significantly earlier, (mean difference −5.53, 95% CI −6.80 to −4.25 days) (17, 18) than those fed by TF. Infants fed with responsive feeding experienced slower weight gain (mean difference −1.36, 95% CI −2.44 to −0.29 g/kg/day) (18–22) but were discharged home earlier than those fed by TF (mean difference −0.48, 95% CI −0.94 to −0.01 weeks) (8, 23). The strategies used in these trials to conduct responsive feeding varied. While most trials used infant cues to initiate and end feedings, the main variation occurred in how and if volume was supplemented after responsive feeding. Specifically, in some studies, if a minimum amount of volume was not taken by oral responsive feeding, a predetermined volume was supplemented by tube feeing. In contrast, in other studies, tube removal occurred upon randomization to responsive feeding and no minimum volume was set. We hypothesized that a modified responsive feeding intervention, which would include: (1) parents as the population conducting the intervention, constituting a parental guided responsive feeding (PGRF); (2) timing and volume of feeds dictated by the infant; and (3) gradual weaning from tube feeding, in which an intermediate volume of food would be supplemented in infants that do not reach a minimum intake, would result in a shortened duration to achieve full oral feeding, earlier discharge home, better weight gain, less apneic episodes and higher parental attendance and engagement in feeding their infants.

## PAtients and Methods

### Inclusion Criteria

A randomized controlled trial was conducted from November 2013 through November 2016 in the neonatal intensive care unit (NICU) of the Chaim Sheba Medical Center in Israel. Preterm infants born prior to 32 weeks of gestation, whose parents signed a written informed consent, were eligible for the study. Exclusion criteria included: birth weight <10th percentile (19) periventricular leukomalacia or intraventricular hemorrhage grade ≥3 on head ultrasound; necrotizing enterocolitis stage ≥2 (20); postnatal steroids; requirement for oxygen support at 33 weeks gestational age (GA); surgery requiring general anesthesia; and proven, suspected, or chronic diseases not associated with prematurity that may have affected the course of hospitalization.

### Randomization

Parents of eligible infants, <33 weeks GA, were approached by one of the study neonatologists (I.M. or T.S.). Randomization was accomplished by the parents taking a sealed opaque envelope from a box. Twins were stratified into the same study group. To ensure balance between the study groups, the envelopes were organized in groups of 8 with an allocation ratio of 1:1, in 10 different boxes numbered 1-10. Although the participants and medical team were not blinded for the study arm assignment, they were blinded for the outcome measures. The study was approved by the Institutional Committee on Human Research and the NIH SHEBA-12-9574-IM-CTIL.

### Settings

At this hospital, ~120 preterm infants are born at <32 weeks each year. Almost all infants are born in hospital and are cared for by the same team from birth until discharge. The NICU is divided into four large rooms, with 10-15 infants in each. Parents are welcome to stay and participate in their infant's care throughout the day, except during nursing shift changes which occur three times a day and take half an hour each time. Parents are provided with chairs and removable curtains for privacy. Most of the parent population live in close proximity to the hospital, resulting in at least one parent being present daily.

Since 2012, the NICU has been transitioning to the Neonatal Individual Developmental Care and Assessment Program (NIDCAP), based on the Synactive theory proposed by Als (21, 22, 24). According to this theory, throughout their development, infants are biologically striving toward self-regulation of increasingly complex abilities. Caregivers can support this emerging competence by attentively and knowledgeably responding to each individual infant's autonomic neurophysiology, behavioral state, and motor (or movement) behavior, so that the infant remains functionally organized and self-regulated.

### Transitioning to Oral Feeding

Gavage trophic feeding is usually initiated during the first day of a preterm infant's life. Mothers' own breast milk is prioritized. Since donor breast milk is not available in Israel, if breast milk is not available, preterm infant formula is provided. Breast milk-fed preterm infants are supplemented with fortified human milk upon reaching 100 ml/kg/day of enteral feeding. The enteral feeding volume is increased by increments of 20–30 ml/kg/day, up to a total daily intake of 140–160 ml/kg/day. The traditional units' guidelines prior to the study period were strict in terms of GA at initiation of oral feeding, intervals between feedings and taken volume, so that direct breastfeeding could be initiated upon arrival to 33 weeks GA. Bottle feeding could be initiated at 34 weeks GA in infants who reach a minimal weight of 1750 g and did not require invasive mechanical ventilation, intervals between feedings were 3 h and given volumes were 140–160 ml/kg/day divided into eight meals. The nasogastric tube was removed at the nurse's discretion, when the infant was perceived to be able to finish the bottle. Infants could be orally fed by parents or nurses. Feeding practices have not been changed in this NICU for more than a decade. Discharge criteria from the NICU included: reaching 36 weeks GA, persistent weight gain, a minimal weight of 1.9 kg, stable temperature, no apneic episodes, and oral feeding ability.

### Study Intervention

Parents whose infants were randomized to the intervention group were asked to participate in a short (up to 60 min) workshop conducted by one of the authors (I.M.), who is trained in NIDCAP. This workshop aimed to provide information and guidance for this intervention, based on current literature (see Supplementary Data 1) (10, 22, 25–28). The workshop introduced parents to the importance of keeping the transition to oral feeding as pleasurable as possible, and avoid intrusiveness, using their infant's cues for guidance. Parents were encouraged to initiate oral feedings within a time span of 2–4 h when signs of hunger were noticed and to terminate feedings when signs of satiety were noticed, regardless of the volume taken. Parents were reassured that if a minimum volume was not taken, supplementation to 65–75% of the maximum would be given via a feeding tube, to prevent weight loss. The intervention started upon arrival to 34 weeks gestation. In order to avoid parental stress and frustration, we did not explicitly recommend parental presence during feedings, however the importance of consistency in caregiver- infant relationship was discussed as part of sensitive care. The minimum required volume of feeding was 90 ml/kg/day, up to a maximum of 180 ml/kg/day, as calculated for six feedings over 24 h. If the infant was fed more frequently, adjustment and re-calculation based on the aforementioned volumes were recommended. If the infant did not achieve a minimum amount per oral feed, or did not arouse after 4 h and no signs of hunger were recognized, an intermediate volume of 120 ml/kg/day over six feeds (20 ml/kg/feed) was supplemented via nasogastric tube. The rationale for this unique strategy was to avoid malnutrition in infants who did not reach a minimum feed, while still allowing a hunger-satiety cycle to develop. The intervention started at 34 weeks GA, as the NICU protocol did not allow for an earlier initiation of oral bottle feeding. The nasogastric tube was removed at the nurse's discretion, when the infant was perceived to be able to finish the minimum volume from the bottle. During the first 2 days of the intervention, if more than 3 h elapsed between feedings, blood glucose (dextrostix) was tested just prior to the next feeding in order to detect hypoglycemia. The intervention continued until the infant was discharged home. A speech therapist, qualified in early infant feeding skills, was hired for the purpose of the study. The speech therapist or the NIDCAP-trained investigator (I.M.) was in attendance during the first day of the intervention, and as needed subsequently. The study protocol, rationale, and the intervention medical orders were introduced to the medical teams (nurses and physicians) during a short presentation so that when parents were not present to feed, adherence to the technical parts of the protocol could be maintained (see Supplementary Datas 1, 2 for details of the workshop and the intervention).

### Baseline Characteristics

The following data were documented: maternal age, income, academic, and marital status, delivery mode, gestational age, gender, Apgar scores, Clinical Risk Index for Babies (CRIB II score), need for mechanical ventilation, duration of oxygen treatment, time needed to regain birth weight and days of intravenous infusion to and amount of breast milk and direct breastfeeding per day. Infants' medical data were collected from the computerized medical charts (MetaVision-MDSoft) using a specific data collection form.

### Outcome Measures

Our primary outcome was measured on the basis of the number of days needed to achieve full oral feeding. In our unit, the feeding tube is removed upon the nurse's discretion, when the infant is perceived to be able to finish a prescribed volume. If the feeding tube is removed, but then an infant does not consume the prescribed volume, then the tube is reinserted. We hypothesized that PGRF would be associated with an earlier progression to full oral feeding. Secondary outcomes and hypotheses included (a) decreased age at discharge as discharge timing is dictated, among other criteria by the ability to orally feed; (b) improved change in weight gain (measured as g/kg/day starting from prior to the intervention until the 36th weeks), as it allowed satiety hunger cycles to develop; (c) increase in percentage of feedings conducted by parents, as the PGRF would result in more pleasurable experience; and d) less apneas and bradycardia events arising from cue-based interactions, due to better synchrony during feedings.

### Statistical Analysis

Data were analyzed using SPSS statistical software, version 25. PGRF and TF infants were compared by independent sample *t*-test or Mann Whitney test for continuous variables, or chi-square tests for categorical ones. Multivariate analysis with Bonferroni correction was used to calculate the outcome measures using repeated measure analysis with interaction effect. Multiple linear regressions were used to assess prediction of days to full oral feeding and age at discharge. We included in the model universal variables as well as variables that were found to be significantly different between the two groups. A value of *p* < 0.05 was considered statistically significant.

Sample size calculation was based on the main outcome measurement of the days needed to achieve full oral feeding. We calculated that a sample size of 30 infants was needed in each group in order to achieve a power of 90% and a level of significance of 5% (two-sided) for detecting a mean difference of −5.0 ± 1, day which was the matrix found by a previous study that assessed the same outcome (18, 29). A number of 40 infants in each group was planned in order to compensate for possible dropout during the study.

## Results

**Figure 1:**
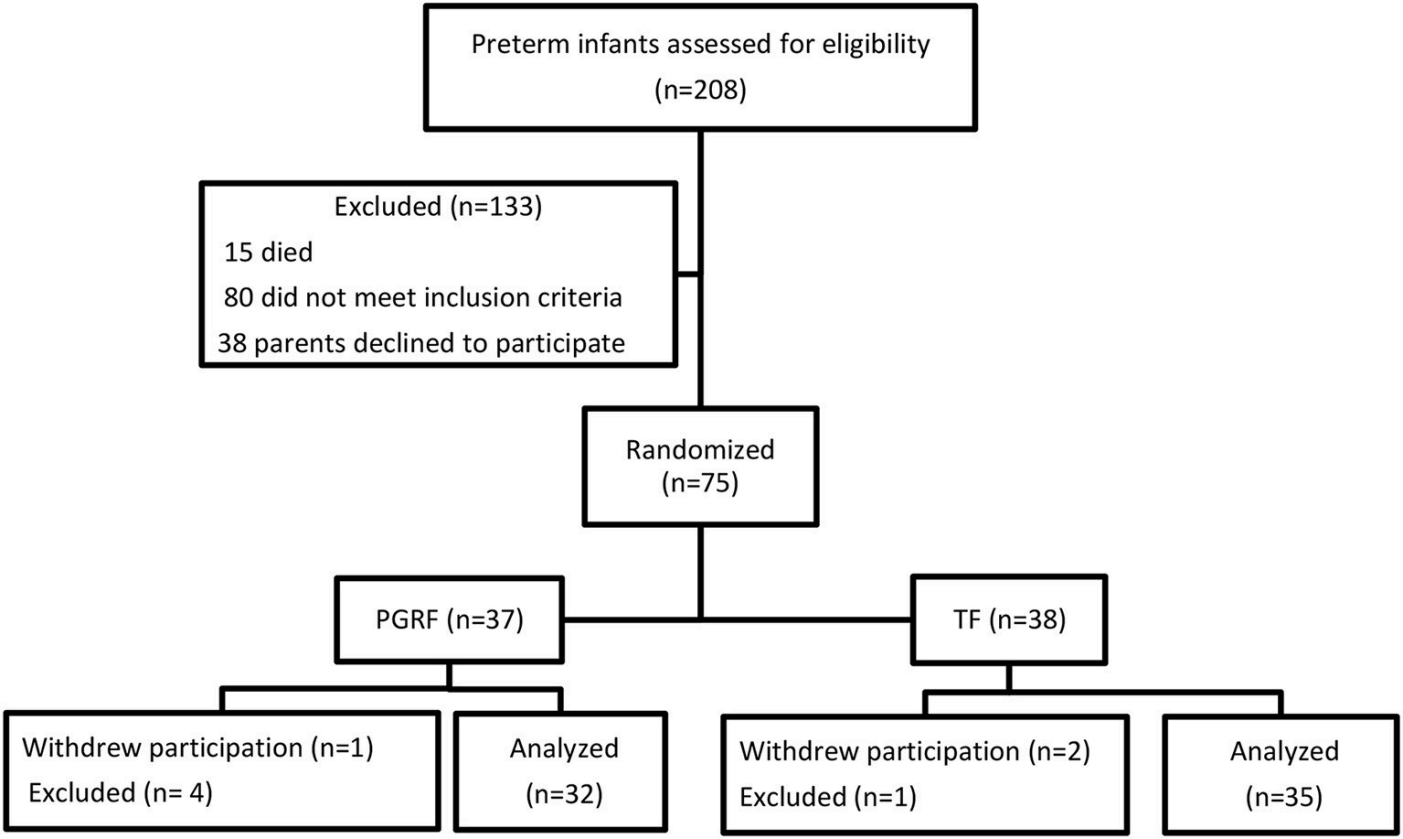
Flowchart of the study participants.

Of the 286 preterm infants born during the study period, 15 died prior to discharge and 80 met the exclusion criteria (Figure 1). The parents of 113 infants were approached; of these, 75 consented to participate. Eight infants were excluded after initial inclusion. Among the excluded PGRF: one required abdominal surgery, one pair of twins was diagnosed with spherocytosis requiring repeated blood transfusions, one was excluded due to maternal illness preventing the mother's presence during transitioning to oral feeding, and one withdrew to return to TF. Among the excluded TF: the parents of a pair of twins withdrew their participation for an unknown reason, and one infant died due to fulminant sepsis. Sixty-seven infants (TF 35, PGRF 32) completed the study and entered the final statistical analysis.

As the study progressed, the investigators noticed that contamination of the study occurred, so that technical strategies used on the PGRF group, such as flexible feeding intervals and the absence of requiring completion of a predetermined volume, were noticed among the TF group. These changes occurred secondary to specific parental or nursing requests. Finally, in November 2016, the nursing teams expressed their frustration at having to feed infants traditionally and requested to change the unit's protocol to PGRF for all. This led to the study being stopped prematurely. Protocol deviation, using PGRF strategies, occurred in 29 (83%) of the controls at a median age of 35.16 weeks (34.28–36.28). Protocol deviation was defined as (i) interval between feedings of less or more than 3 h, or (ii) not completing a predetermined volume of feeding, or (iii) a written medical order that allowed flexibility in timing of feeding or feeding volume.

Table 1 presents the maternal demographics and pregnancy characteristics of the study groups. The groups were comparable in terms of maternal age, marital status, maternal education, rates of *in-vitro* fertilization, surgical delivery, and GA at birth. The following characteristics differed between the groups: (i) being born as part of twins; and (ii) being a first child. The infants' baseline and clinical characteristics were similar among the groups (Table 2).

**Table 1:** Maternal pregnancy and infant demographics and characteristics.

	**PGRF *n* = 32**	**TF *n* = 35**	**Statistic value**	***p*-value**
**MATERNAL CHARACTERISTICS**				
Maternal age (y)	32.03 ± 5.74	33.46 ± 4.89	1.10^a^	0.276
*In-vitro* fertilization	12 (37.5%)	12 (34.3%)	0.75^b^	0.784
First child	24 (75.0%)	17 (48.6%)	4.92^b^	0.027
Prenatal steroids	30 (93.8%)	34 (97.1%)	0.45^b^	0.502
Surgical delivery	21 (65.6%)	27 (77.1%)	1.09^b^	0.296
Gestational age (wks)	29.19 ± 2.27	29.22 ± 2.25	0.04^a^	0.967
Twin	13 (40.6%)	24 (68.6%)	5.28^b^	0.022
Spouse	30 (93.8%)	32 (91.4%)	0.13^b^	0.718
Maternal education (y)	15.63 ± 1.68	15.80 ± 3.09	0.29^a^	0.772
Income				
High	15 (48.4%)	18 (51.4%)		
Middle	13 (41.9%)	11 (31.4%)	1.20^b^	0.548
Low	3 (9.7%)	6 (17.1%)		
**INFANT CHARACTERISTICS**				
Male	16 (50.0%)	19 (54.3%)	0.12^b^	0.726
Apgar 5 minutes	10 (9–10)	10 (9–10)	−0.13^c^	0.897
CRIB II	6 (3–9.75)	6 (5–10)	−1.01^c^	0.313
Birth weight (g)	1279 ± 336	1218 ± 311	−0.77^a^	0.442
Duration of i.v fluids (d)	8 (6–12)	8 (6–14)	−0.07^c^	0.945
Proven sepsis	5 (15.6%)	7 (20.0%)	0.22^b^	0.641
Duration of O2 support (d)	7 (1–23.25)	6 (1–26)	−0.47^c^	0.636
Use of IMV	18 (56.3%)	18 (51.4%)	0.16^b^	0.693
Duration of IMV (d)	1 (0–3)	1 (0–2)	−6.30^c^	0.529

*Data are presented as mean ± standard deviation, median (P25-P75), or n (percent)*.

a *Based on independent sample t-test*.

b *Based on chi-square test*.

c *Based on Mann-Whitney test*.

*PGRF, parental guided responsive feeding; TF, traditional feeding; CRIB II, Clinical Risk Index for Babies; i.v, intra venous; IMV, invasive mechanical ventilation; y, years; wks, weeks; g, gram; d, day*.

**Table 2:** Infant outcomes.

	**PGRF *n* = 32**	**TF *n* = 35**	**Statistic value^a^**	***p*-value**
Weight at 36 weeks (g)	2211 (1982–2393)	2166 (1966–2139)	−0.91	0.363
Breast milk at 34 wks (%)	91.3 (36.05–100)	100 (76–100)	−1.51	0.131
Breast milk at 35 wks (%)	86 (13–100)	100 (43.75–100)	−1.56	0.119
Direct breastfeeding at 34 wks (%)	0 (0–5.32)	5.35 (1–10)	−2.81	0.005
Direct breastfeeding at 35 wks (%)	0 (0–10.80)	6.25 (1.80–16.30)	−2.24	0.025
Apnea/bradycardia at 34 wks	3.5 (1.0–13.50)	9.00 (2.00–18.00)	−1.98	0.047
Apnea/bradycardia 35 wks	1.00 (0–5.75)	3.00 (0–8.00)	−1.44	0.156

*Data are presented as mean ± standard deviation or median (P25-P75)*.

a *Based on Mann-Whitney test*.

*PGRF, parental guided responsive feeding; TF, traditional feeding; g, gram; wks, weeks*.

### Safety

No episodes of hypoglycemia or weight loss that required deviation from the protocol were documented during the study period (34-36 weeks).

### Intervention Effects

**Figure 2:**
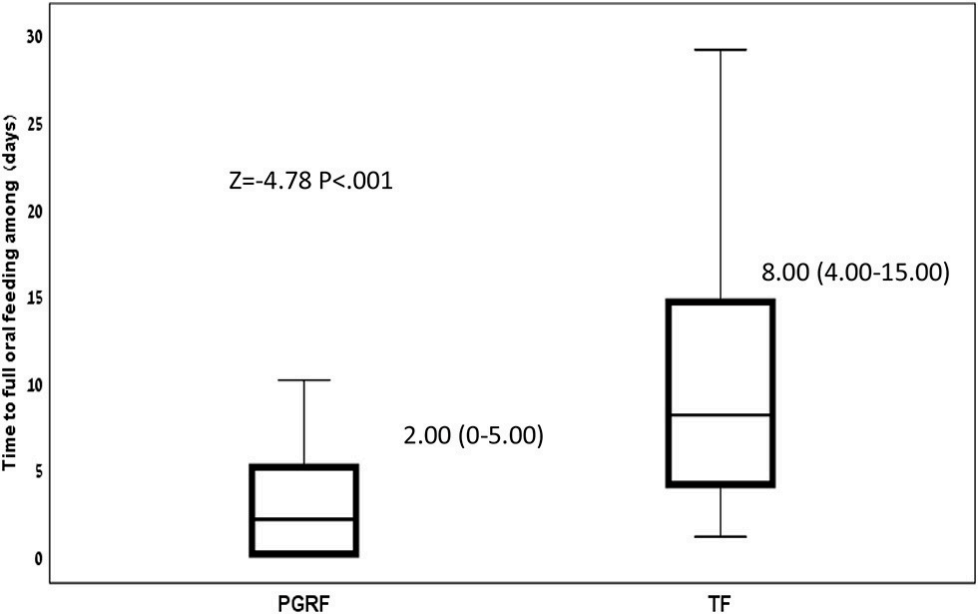
Time to full oral feeding among the study groups. Data are presented as median (P25-P75). Statistical analysis was based on Mann-Whitney-test. PGRF, parental guided responsive feeding; TF, traditional feeding.

**Figure 3:**
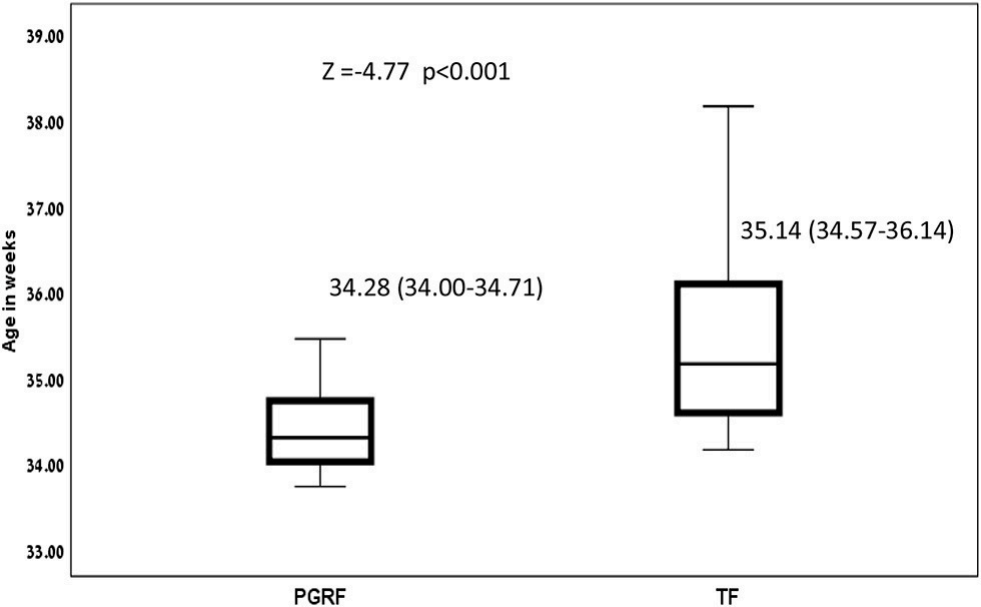
Age at full oral feeding among the study groups. Data are presented as median (P25-P75). Statistical analysis was based on Mann-Whitney test. PGRF, parental guided responsive feeding; TF, traditional feeding.

**Figure 4:**
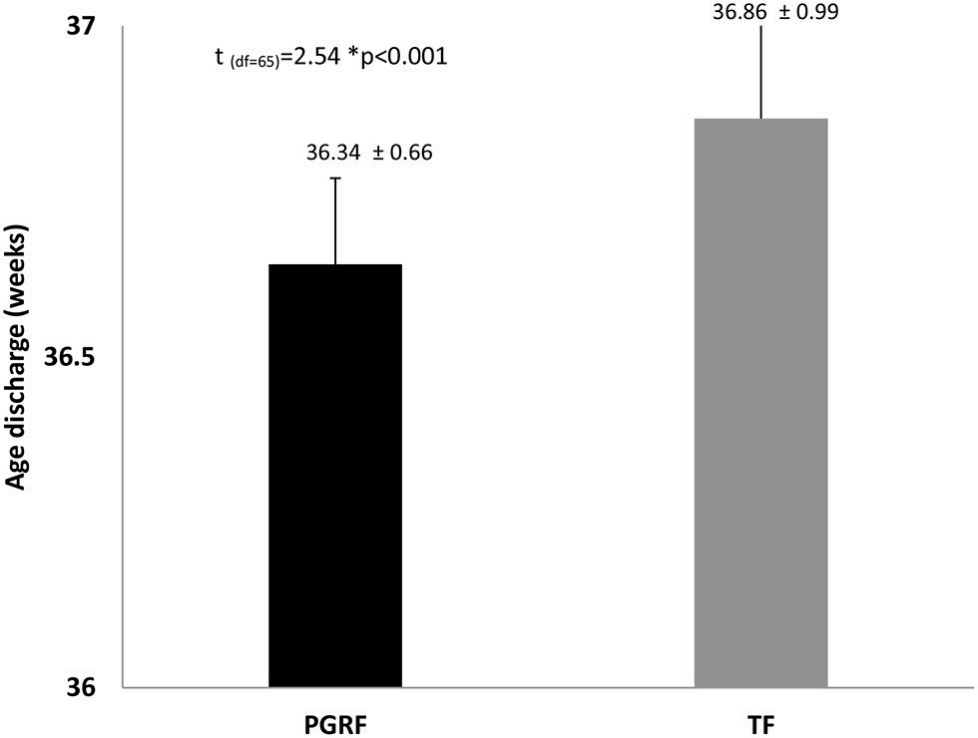
Age at discharge among the study groups. Data are presented as mean ± standard deviation. Statistical analysis was based on independent sample *t*-test. PGRF, parental guided responsive feeding; TF, traditional feeding.

**Figure 5:**
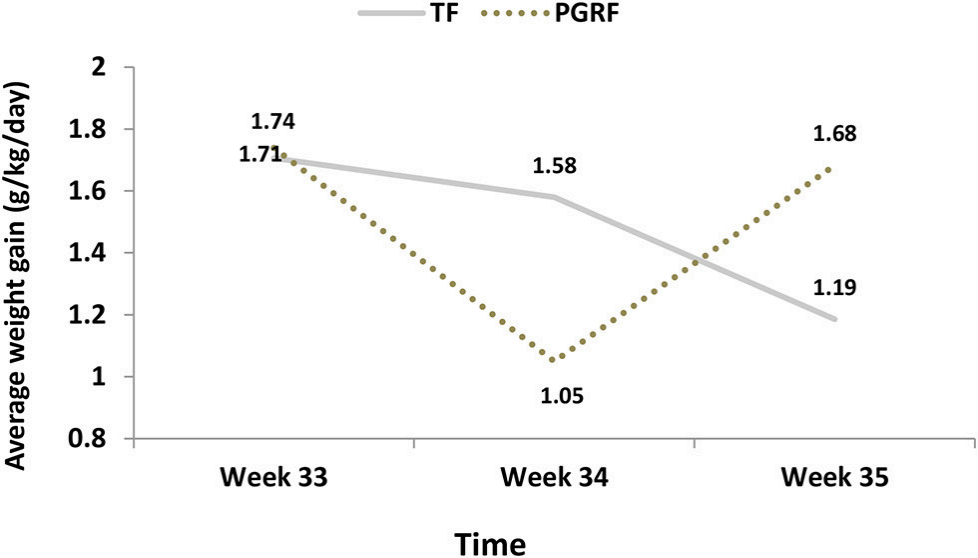
Average weight gain (g/kg/day) among the study groups. PGRF, parental guided responsive feeding; TF, traditional feeding. Analysis was based on repeated measure analysis with Bonferroni correction for time effect. Three main effects were tested: 1. Effect for Time *F*_(2, 126)_ = 7.202 *p* = 0.001, partial eta-squared (ηp2) = 0.103 (10.3%). After Bonferroni correction the significant differences were found between week 33 to week 34 (*p* = 0.002) and week 35 (*p* = 0.023), but not between week 34 to week 35 (*p* = 0.875). 2. Effect for group *F*_(1, 63)_ = 0 *p* = 0.993, partial eta-squared (ηp2) = 0.000 (0%) 3. Interaction effect (Time^*^Group) *F*_(2, 126)_ = 10.921 *p* < 0.001, partial eta-squared (ηp2) = 0.148 (14.8%). [The effect sizes express the amount of variance accounted for by one or more independent variables].

**Figure 6:**
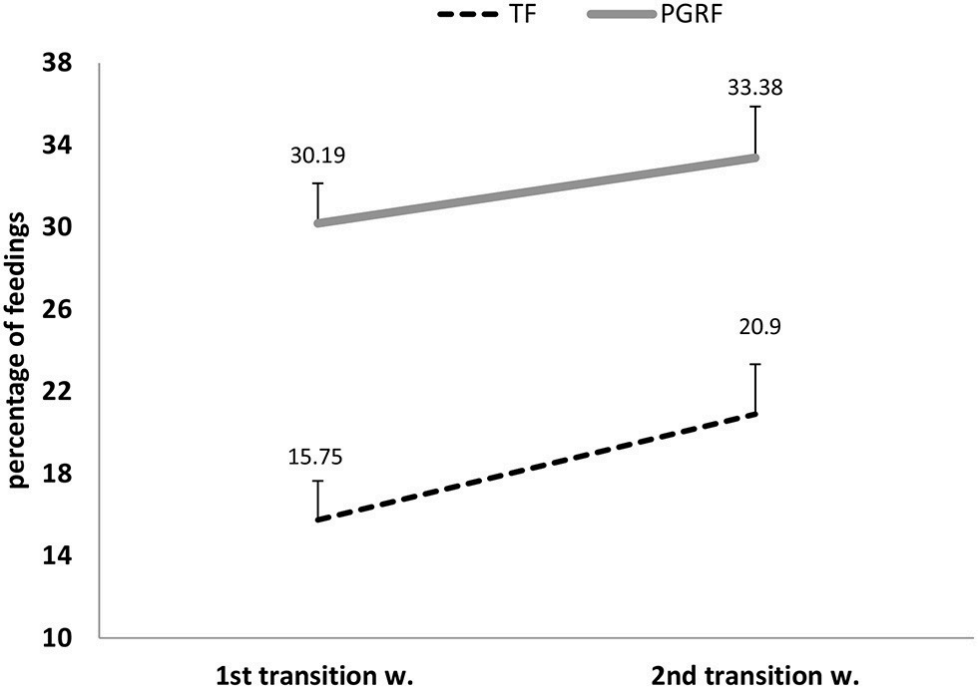
Parental active feeding (percentage) during the intervention among the study groups. PGRF, parental guided responsive feeding; TF, traditional feeding. Analysis was based on repeated measure analysis. Three main effects were tested: 1. Effect for Time *F*_(1, 64)_ = 5.082 *p* = 0.028, partial eta-squared (ηp2) = 0.074 (7.4%). 2. Effect for group *F*_(1, 64)_ = 20.01 *p* < 0.001, partial eta-squared (ηp2) = 0.238 (23.8%). 3. Interaction effect (Time^*^Group) *F*_(1, 64)_ = 0.283 *p* = 0.597, partial eta-squared (ηp2) = 0.004 (0.4%). [The effect sizes express the amount of variance accounted for by one or more independent variables].

Infants randomized to the PGRF group reached full oral feeding significantly earlier (median of 2 vs. 8 days, *p* < 0.001) (see Figure 2) and at a significantly earlier GA (median of 34.28 vs. 35.14 weeks, *p* < 0.001) (see Figure 3). The PRGF group was discharged significantly earlier (mean 36.34 ± 0.66 vs. 36.86 ± 1.00 weeks, *p* = 0.014) (Figure 4). Figure 5 presents the results of the repeated measure analysis of the average weight gain during 33 to 35 completed weeks with the interaction of type of feeding group. We found a significant effect for time [*F*_(2, 130)_ = 7.65 *p* = 0.001, ηp2 = 0.105) suggesting that during the assessed period, the average weight (g/Kg/day) changed significantly. The significant change occurred only between week 33 to week 34 (*p* = 0.002), and between week 33 to week 35 (*p* = 0.009). An interaction effect was observed between time and feeding group [*F*_(2, 130)_ = 9.87 *p* < 0.001, ηp2 = 0.132), meaning that the change in the mean weight gain (g/Kg/day) during weeks 33–36 differed between the groups: while in the TF group, there was a consistent reduction in weight gain between weeks 33–36, in the PGRF group, there was a reduction in the mean weight gain during the first week of the intervention (week 34), which changed into an increase during the second week of the intervention (week 35). This increased rate of weight gain that occurred during week 35 was at a rate similar to the rate of weight gain before the intervention. No significant effect for group was found [F_(1, 63)_ = 0 *p* = 0.993]. Infants who underwent the intervention were significantly more likely to be actively fed by their parents during the intervention weeks compared to the TF group (at 34 weeks GA: 30.19 ± 13.24 vs. 15.75 ± 11.87 percent of feedings, *p* < 0.001; at 35 weeks GA: 33.38 ± 17.01 vs. 20.9 ± 14.70 percent of feedings, *p* < 0.001) (Figure 6).

There was no difference in the percent of breast milk given during the intervention between the groups, although direct breastfeeding was less likely to occur among the PGRF compared to the TF group. Fewer apnea/bradycardia episodes occurred at 34 weeks in the PGRF group compared to the TF group (median 3.5 vs. 9 *p* = 0.047).

In order to predict the time to full oral feeding, a linear regression was conducted. The following independent variables were included in the model: sociodemographic (maternal age and education, spouse, income, being first child, part of twins), infants' characteristics (GA, duration of oxygen treatment) and feeding group. Independent variables predicted 43.7% of the variance of time to full oral feeding [*F*_(9, 65)_ = 4.84 *p* < 0.001]. The only significant variable was feeding group (B = -6.43 *p* < 0.001); The PGRF infants were more likely to reach full oral feeding earlier.

In order to predict age at discharge, a linear regression was conducted. The same independent variables were entered to the model. The result shows that the independent variables predict 22.9% of the variance of age at discharge [*F*_(9, 65)_ = 1.84 *p* = 0.08]. The only significant variable was feeding group (*B* = -0.495 *p* = 0.036); infant feeding in PGRF compare to TF were more likely to be discharged earlier.

## Discussion

The present study sought to investigate the short-term effects of a unique feeding intervention for transitioning preterm infants to oral feeding. This intervention, which was parent-guided and required responsive interaction between the parents and their infants, was proven to be safe. Moreover, infants who underwent the intervention reached full oral feeding within a shorter period of time and at a significantly earlier GA, demonstrated an earlier return to their baseline rate of weight gain prior to the transition from tube to oral feeding, experienced less apnea/bradycardia events during the first intervention week and were discharged home significantly earlier. Increasing demands by parents and the nursing teams to utilize the intervention strategies in all infants resulted in premature termination of the study. This led to generalizing the intervention for all hospitalized infants, suggesting the powerful effect of this feeding strategy on parental (and nursing) perception of infant wellbeing.

In this study, which to the best of our knowledge is the first to target parents as the primary providers of the responsive feeding intervention, we found that the intervention group parents were significantly more likely to attend feedings and feed their infants by themselves. Parents were not directly encouraged to attend feedings; however, the importance of consistency in the care of an infant by its primary caregiver was discussed during the workshop. This finding may suggest higher parental competence and comfort in their infants' care, as well as better engagement. Interventions supporting parents in their skills to observe and interpret their infants' behavior have been associated with improved cognition later in the infants' life (30, 31). A recent longitudinal study that followed preterm infants into adulthood, demonstrated, that good early parent infant relationship defined as frequency of visits, pleasure in the care of their infant and more confidence in their caring practices, predicted an ~5-point increase in adult IQ (32). Such interventions, i.e., that support parental engagement and competence may restore and normalize the parent–infant relationship, promote sensitive and consistent parent-infant interaction and strengthen innate resilience in both parent and child (33). Our results should be interpreted with caution, as we did not directly measure parental satisfaction, nor did we directly measure levels of stress, competence, or the ability to responsively feed. The positive effect could be only hypothesized by the need to stop the study prematurely and expand this intervention to all NICU infants as requested by parents and nurses. Recently, Thoyre et al. studied a novel strategy to co-regulate feeding by mothers (28). The authors stated that major obstacles in delivering the intervention were “inconsistency in the care and divergent ideas,” which were perceived by parents as critical for the success of the intervention. Our experience over the 3 years of this study strongly supports the above finding. Given the many complexities and factors involved in feeding a preterm infant, this process can be made even more difficult when providers are not in agreement regarding the best methods and approaches for doing so (34). The current results indicate that the key to success involves collaborating with parents, maintaining transparency, and establishing consistency.

Being free of nasogastric tubes is a critical milestone in many NICUs for discharge home. Not only does tube removal provide the infant with more comfort and eliminate the burden and stress associated with its recurrent placement, it also allows more freedom for the parents while caring for their hospitalized child. In the present study, preterm infants allocated to the PGRF group reached full oral feeding at a significantly earlier GA and within a significantly shorter period of time. Only two studies to date, both by McCain et al. reported the age of full oral feeding among preterm infants exposed to responsive feeding intervention. Both of these studies differ from the present one in their inclusion criteria and responsive feeding strategies (17, 18). In the first study by McCain et al. the time point for initiating the intervention was 32–34 weeks GA, as compared to 34 weeks GA in the current study. In addition, if a minimum was not achieved, a full amount was supplemented via nasogastric tube, as compared to the intermediate volume that was used in the current study (18). The second study of McCain included extremely premature infants (mean GA 25 weeks) diagnosed with bronchopulmonary dysplasia, unlike the current study in which all infants were free of oxygen support at 33 weeks GA. Furthermore, the intervention was started at a later GA (mean GA 35-36 weeks) (18). In spite of these differences, in all three studies (McCain's studies and the present study), the intervention shortened the time needed to achieve full oral feeding, with a mean of 5 ± 4.2 days and 5.9 ± 4.6 days in McCain's studies and 3.06 ± 3.09 days in ours. We speculate that decreasing the time to achieve full oral feeding in the present study can be attributed to the uniqueness of the intervention i.e., allowing infants to develop hunger satiety cycles using a low minimal volume, avoiding maximal volume supplementation if the minimum was not achieved and allowing up to 4 h between feedings. Of note, the effect of the intervention was preserved when taking into account socioeconomic measures (having a sibling, maternal education, and parental income), indicating that the intervention may be effective in a wide array of populations.

Early postnatal growth has been shown to correlate with long-term outcomes of preterm infants (35). Theoretically, allowing preterm infants to dictate the timing and duration of enteral feeding allows longer rest periods between some feeds and promotes infant-determined sleep and wake patterns, thus reducing unnecessary energy expenditure and increasing growth rates (36). In the present study, infants allocated to the PGRF intervention continued to gain weight throughout the study. Although a reduction in mean weight gain occurred during the first intervention week, it transitioned and returned to the baseline rate during the 2 week. This weight gain pattern was different than the TF group, whose weight gain continued to decline over the study period. The results of a Cochrane meta-analysis suggest that in four trials that included 305 participants slower rate of weight gain was noticed among responsive fed infants (mean difference of −1.36 g/kg/day lower weight gain, 95% CI −2.44 to −0.29 g/kg/day; *I*^2^ = 5%) (8, 16–18, 23). In three of these trials the outcomes were assessed for 10–14 days (8, 18, 23), when ready to be discharge home, while in one study for 1 week only (17). In the present study, we assessed the change in wait (g/kg/day) starting prior to the intervention and continued for 2 weeks. The PGRF group had a significantly slower in weight gain during the first week of intervention however by the 2 week, the rate of weight gain returned to baseline while the TF infants a continues decrease was noted. We speculate that premature removal of the nasogastric tube (upon initiation of the intervention), as occurred in two studies (8, 37), premature assessment of weight outcomes (upon arrival at full oral feeding) (37), and not using an intermediate volume strategy when the minimum volume was not orally reached, may contribute to this difference in results. The present study results are in agreement with the conclusions of Puckett et al. which illustrated the occurrence of a transition time: “2 to 3 days on average are needed to show consistent weight gain” (8). We recommend that caregivers and parents be informed about a transition period in in which weight gain initially slows down, but subsequently returns to a steady rate, similar to the rate of weight gain prior to the feeding transition from tube to oral. We also point to the importance of using intermediate volume supplementation and removal of the nasogastric tube upon infant readiness, rather than according to a pre-planned schedule.

Our model demonstrates only the type of intervention is associated with the duration needed to reach full oral feeding and with age at discharge home. Delay in acquiring feeding skills is the most frequent cause of prolonged hospitalization in the NICU (38, 39). Discharge criteria also include weight (>1900 g), reaching 36 weeks GA, and being free of apnea and bradycardia spells. We speculated that PGRF will result in a significant decrease in apnea and bradycardia frequency. During the first intervention week, apnea/ bradycardia events occurred significantly less than in the PGRF group. Although the frequency of these events continued to decrease during the second intervention week, the rate of apnea/bradycardia events did not reach significance in the second week. This may be explained by physiologic maturity which occurs with increased GA, and which would affect both groups. Yet, the effect of some of the TF infants being fed using PGRF strategies, which was documented among the TF group at 35 weeks GA, cannot be ruled out as the cause for the absence of significance between the groups during the second intervention week.

Only a few randomized studies to date have evaluated the effect of responsive feeding on very preterm infants' outcomes (8, 17, 18, 23, 37, 40–42). Compared to the aforementioned previous studies, this study used a relatively larger number of participants and a unique methodology, as described above.

This study is not free of limitations, the major one being the inability to blind for the intervention, mainly due to the different medical feeding orders in the infants' charts. We assume that this limitation is restricted, as the outcomes measured i.e., time to full oral feed, age at discharge, weight changes etc. were blinded to the medical teams caring for the infants who were making the clinical decisions or documenting in the medical charts. Also, data were retrieved by a different team, which included a non-medical group, thus limiting their influence on medical decisions. A second limitation was the increased use of “intervention strategies” among the controls, creating a contamination in the control group. This, however, occurred at a later GA and involved mainly technical aspects of the intervention (allowing longer time intervals between the feedings and not having to finish the bottle). In spite of the above, the beneficial effect of the intervention could still be demonstrated compared to the TF group, in terms of earlier age at full oral feeding, parents of weight changes over time, and higher parental attendance during feeds, thus supporting the importance of parental responsiveness and early initiation (34 weeks). The third limitation is the recruitment of relatively healthy preterm infants. However, this created a more homogeneous group, and minimized potential confounding factors. Also, once proven safe in healthy preterm population, this intervention can be studied on high risk infants. In spite of these limitation, the significant differences in outcomes between the groups support the powerful effect of this intervention. The fourth limitation is associated with the differences in the demographic characteristics between the groups, such that those infants in the PGRF group were more likely to be first born and significantly less likely to be part of a twin pregnancy. These differences were accounted for in the regression model. Lastly, our cue-based intervention was limited by our traditional protocols, which did not allow initiation of oral feeding prior to 34 weeks or according to infant readiness. We assume that initiation of oral feeding could have been started earlier for the more neurologically mature infants, possibly demonstrating a larger effect in terms of time to full oral feeding and discharge home.

## Conclusion

Our results have some important clinical implications. In particular they emphasize that parental guided cue-based care is feasible and when used for transitioning preterm infants to oral feeding, this intervention significantly shortened the duration needed to achieve oral feeding and discharge home, without compromising weight gain, as measured at 36 weeks GA. Parental presence increased in the PGRF group, which may suggest an increased level of parental competence and confidence. Long-term follow-up assessing developmental outcomes, mother-child interaction, and feeding habits is currently underway.

## Summary

This RCT represents a unique strategy of transitioning preterm infants from nasogastric feeding to oral feeding. This strategy has shown improved short-term outcomes.

## What's Known on this Subject

Few studies to date have evaluated the effect of responsive feeding interventions on preterm infants' outcomes. In these studies, the outcomes demonstrate that responsive feeding can reduce transition time to oral feeding but can be associated with slower rate of weight gain.

## What this Study Adds

In this randomized controlled trial, preterm infants were transitioned to oral feeding using a parental guided responsive feeding strategy. Infants in the intervention group required less time to transition to full oral feeding, gained more weight, were discharged earlier, and were more likely to be fed by their parents.

## Ethics Statement

This study was carried out in accordance with the recommendations of Helsinki Guidlines, according to the Sheba Medical Center Ethics committee with written informed consent from all subjects. All subjects gave written informed consent in accordance with the Declaration of Helsinki. The protocol was approved by the Sheba Medical Center committee.

## Author Contributions

IM conceptualized and designed the study, coordinated and supervised data collection, analyzed the data and drafted the initial manuscript, and reviewed and revised the manuscript. YH coordinated and supervised data collection, co-analyzed the data and revised the manuscript. DK helped in reviewing the literature, drafted and reviewed the manuscript. RG conceptualized and designed the study, conducted all data analyses, drafted the initial manuscript, and reviewed and revised the manuscript. ST co-analyzed the data, reviewed and revised the manuscript. All authors approved the final manuscript as submitted and agree to be accountable for all aspects of the work.

### Conflict of Interest Statement

The authors declare that the research was conducted in the absence of any commercial or financial relationships that could be construed as a potential conflict of interest.

## Abbreviations

NGT: nasogastric tube
NICU: Neonatal Intensive Care Unit
GA: gestational age
PGRF: parental guided responsive feeding
TF: traditionally fed
hTF: historical group
GA: gestational age
BW: birth weight
NICU: neonatal intensive care unit
NIDCAP: Neonatal Individual Developmental Care and Assessment Program
CRIB II: Clinical Risk Index for Babies.
