# Supplementary material for: Transition From Nasogastric Tube to Oral Feeding: The Role of Parental Guided Responsive Feeding
Source: Front Pediatr. 2019 May 9;7:190. doi: 10.3389/fped.2019.00190 (PMC6521795; doi:10.3389/fped.2019.00190)
Supplement: Supplementary file 1 [file Data_Sheet_1.docx]

Parental guided responsive feeding workshop for parents:

1) **Theoretical section** in which parents were introduced to the physiological and psychological importance of cue-based interaction (1). In this context, feeding quality (i.e., infant’s ability to maintain its physiologic stability and parent’s ability to identify and appropriately respond to an infant’s cues during a feeding) as opposed to feeding quantity was used as an example (2). Mutual joy and pleasure, beginning with the first oral feeding, was as the main target, as opposed to placing emphasis on the technical aspects of feeding (3-5).

2). **Practical strategies section** on how to provide PGRF based on relevant published literature (6,7). The following domains were discussed: **i)** Recognizing an infant’s readiness to feed, such as being awake and demonstrating signs of hunger (moving extremities or head, mouthing, rooting or sucking) as well as signs of satiety (sleep or failure to maintain sucking). **ii)** Signs of dysregulation and stress (repeated and frequent change in skin color or breathing pattern, apnea, bradycardia, change in muscle tone, stretching or arching, coughing or choking). **iii)** Strategies for supporting and positioning the infant during the feeding i.e; cradled with the head at midline in a neutral position, while extremities are supported and hands flexed at midline, or side-lying, or semi-upright in front. **iv)** External pacing of suck-swallow-breath coordination (rate of 3-10 :1 sucking bursts: breathing pause ) was encouraged. **v**) Lastly, intrusive behavior, forced oral feeding, facial or bottle manipulations, oral stimulation, face massage, and bottle turning were discouraged (7-9).

**3). Joint video observations** of feeding interactions: The parents watched videos of feeding interactions and were asked to identify the feeding strategies that were used and the infants' cues. Questions and discussions were encouraged.

**4) Introducing the PGRF medical format section** (Figure 1), which included presentation of the new computerized medical feeding orders. In general, we recommended that a feeding should not last longer than 30 minutes and that oral feeding should be stopped if signs of dysregulation or compromise were noticed.

Figure 1. PGRF medical orders for an 1800 grams infant:


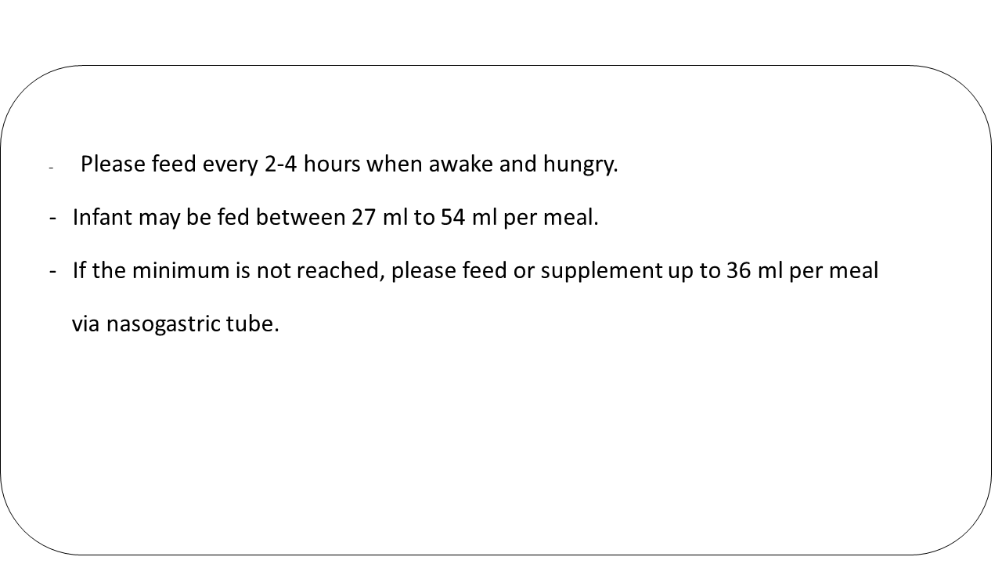


*The nurses were asked to follow the above medical orders, regardless of parental presence.

**References:**

1. Als H. Toward a synactive theory of development: Promise for the assessment and support of infant individuality. *Infant Ment Health J.* 1982 Dec 1;3(4):229–43
2. Ross ES, Philbin MK. SOFFI: An evidence-based method for quality bottle-feedings of preterm, ill, and fragile infants. *J Perinat Neonatal Nurs.* 2011;25(4):349–59
3. Pridham K, Lin CY, Brown R. Mothers’ evaluation of their caregiving for premature and full-term infants through the first year: contributing factors. *Res Nurs Health.* 2001 Jun;24(3):157–69
4. Thoyre SM, Brown RL. Factors contributing to preterm infant engagement during bottle-feeding. *Nurs Res.* 2004 Oct;53(5):304–13
5. Pickler RH. A Model of Feeding Readiness for preterm infants*. Neonatal Intensive Care.* 2004;17(4):31–6.
6. Shaker CS. Cue-based co-regulated feeding in the neonatal intensive care unit: supporting parents in learning to feed their preterm infant. *Newborn and Infant Nursing Reviews.* 2013 Mar 1;13(1):51–5
7. Thoyre SM, Shaker C, Pridham K. Manual For administration of the early feeding skills assessment (EFS). 2009
8. Kirk AT, Alder SC, King JD. Cue-based oral feeding clinical pathway results in earlier attainment of full oral feeding in premature infants. *J Perinatol.* 2007 Sep;27(9):572–8
9. Puntis JWL. Nutritional support in the premature newborn. *Postgrad Med J.* 2006 Mar;82(965):192–8
