# Supplementary material for: Transition From Nasogastric Tube to Oral Feeding: The Role of Parental Guided Responsive Feeding
Source: Front Pediatr. 2019 May 9;7:190. doi: 10.3389/fped.2019.00190 (PMC6521795; doi:10.3389/fped.2019.00190)
Supplement: Supplementary file 2 [file Data_Sheet_2.docx]

**Practical guidelines for Parental Guided Responsive Feeding**

**Assessment prior to initiating oral feeding:**

1. *Is the infant 34 0/7 weeks?*
2. *Is the infant breathing room air and free of respiratory support?*
3. Does the infant weight >1750g*?*
4. *Does the infant tolerate enteral nutrition?*
5. **If all of the above are positive and the parents participated in the PGRF workshop then proceed to oral feeding if:**
6. *The infant shows cues of hunger (rooting, mouthing, hands to mouth)*
7. *The infant is in an awake state*
8. *>2 hours have elapsed since last feed*
9. **If all are positive, proceed to oral feeding while being attentive to the following:**
10. *Support the infant's position (extremities flexed and head in midline)*
11. *Introduce the nipple to the infant while avoid pushing it into the infant mouth*
12. *Use pacing to a rhythm of 3-10 sucking bursts:1 breathing pause*
13. *Respect sucking pauses and avoid prodding*
14. *If breast fed: proceed to breastfeed as per lactation consultant recommendations*

1. **Stay attentive to the infant's cues during feeding. Stop feeding if the following are noticed:**
2. *The infant is disengaged: no active rooting or sucking, or the infant pushed the nipple out of his/her mouth*
3. *The infant falls asleep*
4. *The infant demonstrated increased work of breathing or repeated desaturations*
5. *The infant demonstrates signs of fatigue*
6. **If 2a and/or 2b are negative and four hours have elapsed:**

*a. Gently try to bring the infant to an awake state and continue to step 3*

1. **If the taken volume did not reach the minimum (15 ml/kg/meal) or If the infant cannot be awoken after 4 hours**:
2. *Feed via tube 20 ml/kg/meal*

**During the transition period assess:**

Glucose levels during the first 24 hours of the intervention

Daily volume intake

Weight every 48-72 hours
